# Association Between Occlusion Type and Etiology of Acute Intracranial Large Artery Occlusion

**DOI:** 10.3389/fneur.2020.582388

**Published:** 2020-10-22

**Authors:** Huang Chuming, Hong Yifan, Xu Ke, Xu Chukai, Zhang Weijie, Li Hui, Peng Guoyi, Zhang Xiaoyong, Zhang Peifeng, Cai Chuwei

**Affiliations:** Shantou Central Hospital, Shantou, China

**Keywords:** stroke, thrombectomy, stent, intracranial large artery occlusion, intracranial atherosclerosis

## Abstract

**Objective:** To investigate the diagnostic efficiency of truncal-type occlusion and branching-site occlusion in determining the etiology of intracranial large artery occlusion related acute ischemic stroke (AIS).

**Methods:** Patients with intracranial large artery occlusion related AIS who received stent retriever (SR) thrombectomy from November 2014 to June 2019 were included in the study. All patients underwent angiography before SR thrombectomy, which was used to evaluate the occlusion type. Differences in the distribution of occlusion types in intracranial atherosclerosis (ICAS) and embolism were assessed, and the diagnostic indicators, including the area under the ROC curve (AUC), sensitivity, and specificity were calculated.

**Results:** Of the 115 AIS patients with intracranial large artery occlusion, 42 were classified as having ICAS, and 73 having an embolism. In the ICAS group, branching-site occlusion was responsible for 3 (7%) cases and truncal-type occlusion for 39 (93%) cases, while in the embolism group, branching-site occlusion was responsible for 66 (90%) cases and truncal-type occlusion for 7 (10%) cases; the difference was statistically significant (all *P* < 0.01). The AUC for ICAS predicted by truncal-type occlusion was 0.916, with a sensitivity of 92.86%, and specificity of 90.41%.

**Conclusion:** Truncal-type occlusion showed a high predictability of ICAS. Determine the etiology of intracranial large artery occlusion related AIS before SR thrombectomy may be most helpful in setting up optimal endovascular treatment strategies.

## Introduction

Stent retriever (SR) thrombectomy is a primary treatment for acute ischemic stroke (AIS) patients with intracranial large artery occlusion (1, 2). Nevertheless, SR has been shown to be less effective in AIS patients with intracranial atherosclerosis (ICAS). The ICAS accounts for 15–35% of the total acute intracranial large artery occlusion (3, 4), and the proportion is even higher in the Asian populations (5, 6). The SR thrombectomy was less satisfactory in patients with ICAS-related occlusion (7, 8). Repeated mechanical thrombectomy might cause intima injuries and acute thrombosis, which may subsequently lead to revascularization failure (9). Revascularization for patients with ICAS-related occlusion usually requires balloon dilatation or rescue therapy with stenting (10–12). In contrast, in patients with embolism, repeated SR thrombectomy is often required because of hard-organized clots or location of embolism (13). Regarding the differential revascularization strategies for AIS patients with ICAS and embolic occlusions (14), it is crucial to determine the etiology of occlusion before mechanical thrombectomy in order to improve the strategy for revascularization.

Although the high-resolution vessel wall magnetic resonance imaging (MRI) is useful in diagnosis of occlusion type (15), it is expensive and not readily available. Rely on MRI along may delay the treatment for revascularization. Moreover, it is difficult to determine the occlusion type through the baseline angiography before mechanical thrombectomy. In a study by Baek et al. (16), different occlusion types (branching-site occlusion and truncal-type occlusion) predicted the etiology of acute ILAO. However, the prevalence of truncal-type occlusion in patients with ICAS was only 39.5%, which might be because they substituted embolism and ICAS with and without embolic sources. Asian populations were reported to have higher incidence of intracranial arterial stenosis than the western populations. Patients with intracranial arterial stensis are often complicated with atherosclerotic stenosis of the extracranial artery or the aorta (17). If intracranial arterial stensis is complicated with extracranial arterial stenosis and acute ICAS-related occlusion occurs in the intracranial artery, patients may be misclassified as with an embolic source (16).

Angiographic images obtained from SR thrombectomy have been considered as a valuable method to distinguish ICAS from an embolism source in mechanical thrombectomy (18–21). In our study, angiography images were used to examine the diagnostic efficiency of truncal-type occlusion and branching-site occlusion in determining the etiology of acute intracranial large artery occlusion related ischemic stroke.

## Methods

We performed a retrospective study on AIS patients with intracranial large artery occlusion who received mechanical thrombectomy from November 2014 to June 2019. Inclusion criteria included: (1) intracranial large artery occlusion occurred within 24 h after AIS; (2) intracranial large artery occlusion was diagnosed with angiography; (3) all patients received SR thrombectomy; (4) the occlusion occurred in the intracranial internal carotid artery, the middle cerebral artery M1 or the basilar artery; (5) over 18 years old; (6) the pre-operational MRS score was ≤ 1. Exclusion criteria were as follows: (1) patients with intracranial large artery occlusion caused by one of the following reasons: dissection, vasculitis, and Moyamoya disease; (2) the etiology could not be determined by angiography after SR thrombectomy. All patients underwent mechanical recanalization under local anesthesia, followed by the insertion of 8F sheath through the femoral artery, the insertion of 8F or 6F guiding catheter to the internal carotid artery or vertebral artery, and SR thrombectomy with Solitaire AB/FR (Covidien/ev3, Irvine, CA) embolectomy device. Patients with SR thrombectomy failure underwent balloon dilatation or balloon dilatation combined with rescue stenting, with solitareAB/FR or Enterprise (Codman, Florida, United States).

### Definitions of ICAS and Embolism

**Figure 1:**
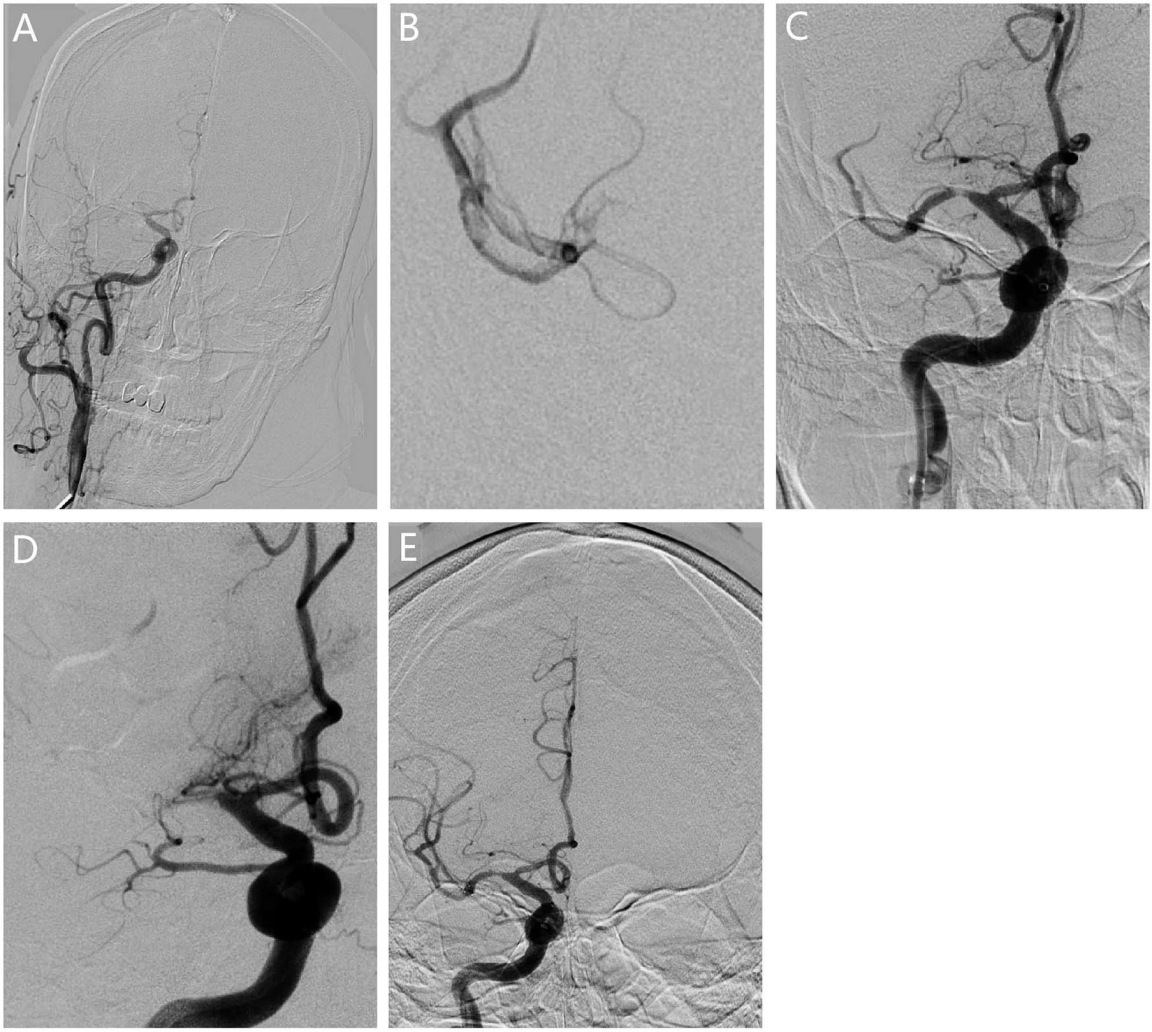
Revascularization for patients with ICAS. **(A)** The angiogram showed occlusion in the right middle cerebral artery M1. **(B)** The microcatheter reached the occlusion segment showing bifurcation of the middle cerebral artery, confirming the truncal-type occlusion. **(C)** Vessel revascularization after SR thrombectomy and residual stenosis. **(D)** The angiography showed that occlusion occurred again 10 min after surgery. **(E)** Rescue therapy with stenting was given for revascularization.

**Figure 2:**
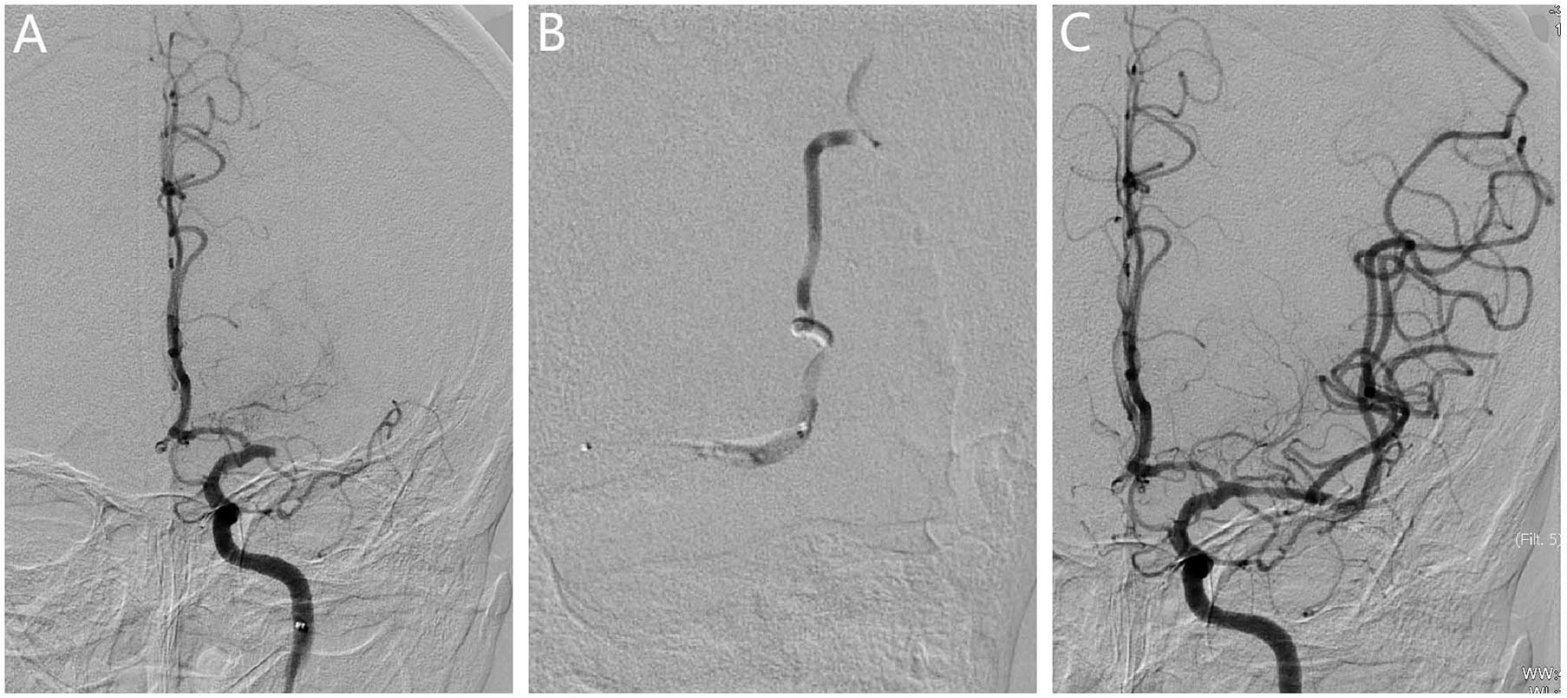
Revascularization for patients with embolism. **(A)** The angiogram showed the occlusion in the left middle cerebral artery. **(B)** The microcatheter reached the occlusion segment, and bifurcation was not observed at the distal end of the occlusion segment, which further confirmed the presence of branching-site occlusion. **(C)** Vessel revascularization after SR thrombectomy and no residual stenosis was observed.

The etiology of intracranial large artery occlusion related AIS was classified into ICAS and embolism according to the angiography after SR thrombectomy. Based on the methods by Lee et al. (18), embolism was confirmed if complete revascularization without obvious residual stenosis was observed on angiography after SR thrombectomy, which was then reconfirmed by CTA or MRA a week later. ICAS was confirmed if any of the following occurred after SR thrombectomy: (1) residual stenosis was >70%; (2) moderate stenosis with flow impairment; (3) CTA and MRA after a week indicating a worsening in residual stenosis or an occurrence of occlusion (Figures 1, 2).

### Definition of Truncal-Type and Branching-Site Occlusions

An intracranial large artery occlusion was classified as the truncal-type if the bifurcation was observed at the distal end of the occlusion based on the countercurrent of contrast agent in the angiogram. An intracranial large artery occlusion was classified as the branching-site occlusion if one of the following criteria were met: (1) bifurcation was not observed at the distal end of the occlusion segment; (2) if the occlusion occurred in the intracranial internal carotid artery, and the middle cerebral artery proximal to the occlusion was not observed in the contralateral angiograph or the microcatheter-mediated angiography showed a contra-flow, it showed that the occlusion persisted into the middle cerebral artery M1.

The SR thrombectomy was considered to be successful if the following conditions were achieved: the forward blood flow was recanalized after no more than three SR thrombectomies, with mTICI grade 2c or 3; if stenosis was still observed 10 min post-surgery; the forward flow was not slowed down nor did the remaining stenosis get worse. SR thrombectomy was considered as failure if the blood flow remained unrecovered or patients presented with occlusion within 5 min after the SR thrombectomy. Two experienced neurointerventionalists examined all the angiograms.

### Statistical Analysis

All analyses were performed using SPSS19.0. Continuous variables were examined by independent two-sample *t*-tests, and categorical variables were examined by Chi-square tests. We plotted ROC curves to assess the diagnostic parameters, giving area under the ROC curves (AUCs) and 95% confidence interval (CIs). Sensitivity, specificity, accuracy, positive predictive value, negative predictive value, positive likelihood ratio, and negative likelihood ratio were also calculated. A two-sided *P* < 0.05 was considered statistically significant.

## Results

### Baseline Information

Of 115 patients in this study, 42 patients were classified as having ICAS (36.5%) and 73 as having embolism (63.5%). The patients with ICAS had a lower prevalence of atrial fibrillation, higher levels of LDL-C, and higher prevalence of balloon dilatation and rescue stenting than patients with embolism (*P* from < 0.001 to 0.008). No significant difference in age, gender, prevalence of hypertension, diabetes, forward flow with mTICI grade 2c or 3 and acute stent occlusion was observed. Acute stent occlusion is defined as stent occlusion revealed by CTA/MRA within 1 week after stent implantation (Table 1).

**Table 1:** Baseline information of patients with ICAS and embolism.

	**ICAS (*n* = 42)**	**Embolism (*n* = 73)**	***p*-value**
Age, mean (SD)	62.10 (11.73)	64.08 (13.52)	0.429
Gender, (M/F), *n*	25/17	51/22	0.259
Hypertension, *n* (%)	31 (73.8)	509 (68.5)	0.547
Diabetes, *n* (%)	13 (31.0)	17 (23.3)	0.367
Atrial fibrillation, *n* (%)	2 (4.8)	28 (38.4)	<0.001
LDL-c, (mmol/L), mean (SD)	2.91 (0.99)	2.42 (0.89)	0.008
**Location of occlusions**, ***n*** **(%)**			
Intracranial internal carotid artery	4 (9.5)	29 (39.7)	
Middle cerebral artery M1	32 (76.2)	34 (46.6)	
Basilar artery	6 (14.3)	10 (13.7)	
Balloon, *n* (%)	34 (81.0)	17 (23.3)	<0.001
Rescue stenting, *n* (%)	34 (81.0)	18 (24.7)	<0.001
Mtici 2c/3, *n* (%)	40 (95.2)	63 (86.3)	0.233
Acute stent occlusion, *n* (%)	2 (4.8)	3 (4.1)	1.000

### The Incidence of Occlusion Types in Different Etiologies

Table 2 shows that, of the 115 patients with AIS due to intracranial large artery occlusion, 3 (7.1%) of the 42 patients with ICAS had branching-site occlusion while 39 had truncal-type occlusion; 66 (90.3%) of the 73 patients with embolism had branching-site occlusion while 7 had the truncal-type occlusion. The difference was statistically significant (*P* < 0.001). Similar distribution of occlusion types was also found in the intracranial internal carotid artery, middle cerebral artery M1, and the basilar artery between the patients with ICAS and embolism (P from <0.001 to 0.001) (Table 2).

**Table 2:** The distribution of occlusion types in different arteries, *n* (%).

		**ICAS**	**Embolism**	***P*-value**
Intracranial artery	Truncal-type	39 (92.9)	7 (9.6)	<0.001
	Branching-site	3 (7.1)	66 (90.3)	
Intracranial internal carotid artery	Truncal-type	3 (75)	0 (0)	0.001
	Branching-site	1 (25)	29 (100)	
Middle cerebral artery M1	Truncal-type	30 (93.8)	6 (17.6)	<0.001
	Branching-site	2 (6.2)	28 (82.4)	
Basilar artery	Truncal-type	6 (100)	1 (10)	0.001
	Branching-site	0 (0)	9 (90)	

### Diagnostic Efficiency

The AUC of the patients with ICAS diagnosed by truncal-type occlusion in the intracranial artery was 0.916 with a sensitivity of 92.86%, a specificity of 90.41%, a positive predictive value of 84.79%, a negative predictive value of 95.65%, an accuracy of 91.30%, a positive likelihood ratio of 9.68, and a negative likelihood ratio of 0.08. The efficacy of the patients with ICAS diagnosed by truncal-type occlusion in the intracranial internal carotid artery, the middle cerebral artery M1, and the basilar artery is shown in Table 3.

**Table 3:** Diagnostic efficiency of ICAS by truncal-type occlusion.

	**Sensitivity %**	**Specificity %**	**Accuracy %**	**PPV**	**NPV**	**PLR**	**NLR**	**AUC (95%CI)**
Intracranial artery	92.86	90.41	91.30	0.848	0.957	9.68	0.08	0.916 (0.856–0.976)
Intracranial internal carotid artery	75	100	97	100%	0.967	NA	0.25	0.875 (0.616–1.134)
Middle cerebral artery M1	93.75	82.35	87.8%	0.833	0.933	5.31	0.08	0.881 (0.790–0.971)
Basilar artery	100	90	94	0.857	1	10	NA	0.950 (0.834–1.066)

*ICAS, intracranial atherosclerosis; PPV, positive predictive value; NPV, negative predictive value; PLR, positive likelihood ratio; NLR, negative likelihood ratio; AUC, area under curve; CI, confidence interval; NA, not available*.

### Revascularization

Among the patients with truncal-type occlusion, 91.3% were grade 2c or 3 in the final mTICI grade, while 88.4% of the patients with branching-site occlusion were graded 2c or 3. There was no significant difference between the two groups. However, the patients with truncal-type occlusion showed higher rate of balloon (71.7% vs. 26.1; *P* < 0.001) and rescue therapy with stenting (69.6% vs. 31.9%; *P* < 0.001; Table 4).

**Table 4:** Clinical characteristics by occlusion type.

	**Truncal-type (*n* = 46)**	**Branching-site (*n* = 69)**	***P*-value**
Age, mean (SD)	62.22 (11.71)	64.12 (13.63)	0.442
Gender, (M/F), *n*	29/17	43/26	0.937
Hypertension, *n* (%)	34 (73.9)	46 (66.7)	0.408
Diabetes, *n* (%)	13 (28.2)	16 (23.2)	0.539
Atrial fibrillation, *n* (%)	2 (4.3)	28 (40.6)	<0.001
LDL-c, (mmol/L), mean (SD)	2.81 (0.94)	2.46 (0.95)	0.059
**Location of occlusions**, ***n*** **(%)**			
Intracranial internal carotid artery	3 (6.5)	30 (43.5)	
Middle cerebral artery M1	36 (78.3)	30 (43.5)	
Basilar artery	7 (15.2)	9 (13.0)	
Balloon, *n* (%)	33 (71.7)	18 (26.1)	<0.001
Rescue stenting, *n* (%)	32 (69.6)	22 (31.9)	<0.001
Mtici 2c/3, *n* (%)	42 (91.3)	61 (88.4)	0.852
Acute stent occlusion, *n* (%)	2 (4.3)	3 (4.3)	1.000

## Discussion

Our study showed that classification of occlusion type was highly valuable in the diagnosis of intracranial large artery occlusion related AIS, because it is more likely for a floating clot to stick to the bifurcation where the blood vessel suddenly narrows and sharply turns compared the trunk of the middle cerebral artery M1 or basilar artery where the blood vessel is straight, and there is little change in the diameter at the distal and proximal end. Meanwhile, most of the intracranial arterial stensis occurs in the trunk of the artery. Of note, previous studies also showed that intracranial arteriostenosis preferably occurs in the mid-lower segment of the basilar artery, internal carotid siphon, and the trunks before bifurcation in the M1 segment of the middle cerebral artery (22, 23).

Baek et al. demonstrated that the occlusion types (truncal-type occlusion and branching-site occlusion), were independent predictors for ICAS and embolism in intracranial large artery occlusion related AIS (16). Of 202 patients with branching-site occlusion who received SR thrombectomy, 161 were revascularized (79% success rate), while only 4 out of 22 patients with truncal-type occlusion were revascularized by SR thrombectomy (18% success rate) (16). In patients with truncal-type occlusion, 52% had ICAS, which was represented as without an embolic source, and 48% were with an embolism source. Previous studies (15–17) suggested that SR thrombectomy was not very effective in patients with ICAS-related occlusion, and balloon or rescue stenting therapies are often required for revascularization. On the other hand, the success rate of SR thrombectomy was high in patients with embolismrelated occlusion, which was due to the different disease mechanisms. SR thrombectomy could induce intima injury around the ICAS related occlusion, which activated the platelets and led to occlusion again. In Baek et al.'s study (16), an 18% success rate of SR thrombectomy in patients with truncal-type occlusion was found, suggesting that most patients with truncal-type occlusion might be ICAS-related occlusion. However, nearly half of the patients with truncal-type occlusion were patients with embolism. Such conflicting findings suggested that there might be defects when ICAS and embolism were substituted with the presence and absence of an embolic source.

Angiography after SR thrombectomy has been widely used for the diagnosis of intracranial large artery occlusion relatedAIS (18–21). We used this method to determine the etiology of occlusion and found that of 46 patients with truncal-type occlusion, 39 had ICAS (84.8%) and 7 had embolism (15.2%). The prevalence of ICAS in the truncal-type group was much higher than that reported by Baek et al. (16). After improving the diagnostic method for ICAS and embolism, the diagnostic efficiency of ICAS patients by truncal-type occlusion was also improved substantially, with a sensitivity being 92.86%, specificity being 90.41%, and AUC being 0.916. Moreover, we also compared the diagnostic efficiency of occlusion type in different intracranial arteries and found that occlusion type showed better diagnostic efficiency in basilar artery than in the intracranial internal carotid artery and the middle cerebral artery M1. This may be because arterial stenosis preferably occurs in the proximal end of the basilar artery (24, 25). Furthermore, as the trunk of the basilar artery is straight, while the top of the basilar artery has a higher curvature, the floating clot is likely to stick to the top.

Our study had some limitations. Firstly, the present study was a single-center study, which only included patients in a city in China. Generalizability to the western populations should be examined. In addition, we used the radiography after SR thrombectomy for the etiological diagnosis of occlusion, which is the best indicator in mechanical thrombectomy. However, it is still not the gold standard. Finally, the sample size is not large. However, owing to the comprehensive examination for all patients, our study might have provided the best available evidence for making clinical decisions.

## Conclusion

Classification of occlusion type plays an important role in the etiological diagnosis of occlusion. The truncal-type occlusion predicted the ICAS accurately. Determining the etiology of occlusion before SR thrombectomy is important for development of revascularization strategies in intracranial large artery occlusion related AIS in clinical practice.

## Data Availability Statement

The raw data supporting the conclusions of this article will be made available by the authors, without undue reservation.

## Ethics Statement

The studies involving human participants were reviewed and approved by Shantou Central Hospital medical ethics committee. The patients/participants provided their written informed consent to participate in this study.

## Author Contributions

HC and CC contributed to the conception and design of the study. HY contributed to preparation of the manuscript. XK, XC, ZW, LH, PG, ZX, and ZP contributed to acquisition of data. All authors contributed to the article and approved the submitted version.

## Conflict of Interest

The authors declare that the research was conducted in the absence of any commercial or financial relationships that could be construed as a potential conflict of interest.
